# Nutrition, Epigenetics, and Major Depressive Disorder: Understanding the Connection

**DOI:** 10.3389/fnut.2022.867150

**Published:** 2022-05-18

**Authors:** Miguel A. Ortega, Óscar Fraile-Martínez, Cielo García-Montero, Miguel Angel Alvarez-Mon, Guillermo Lahera, Jorge Monserrat, Maria Llavero-Valero, Fernando Mora, Roberto Rodríguez-Jiménez, Sonia Fernandez-Rojo, Javier Quintero, Melchor Alvarez De Mon

**Affiliations:** ^1^Department of Medicine and Medical Specialities, University of Alcala, Alcalá de Henares, Spain; ^2^Ramón y Cajal Institute of Sanitary Research (IRYCIS), Madrid, Spain; ^3^Cancer Registry and Pathology Department, Hospital Universitario Principe de Asturias, Alcalá de Henares, Spain; ^4^Department of Psychiatry and Mental Health, Hospital Universitario Infanta Leonor, Madrid, Spain; ^5^Psychiatry Service, Center for Biomedical Research in the Mental Health Network, University Hospital Príncipe de Asturias, Alcalá de Henares, Spain; ^6^Department of Legal Medicine and Psychiatry, Complutense University, Madrid, Spain; ^7^Institute for Health Research 12 de Octubre Hospital, (Imas 12)/CIBERSAM (Biomedical Research Networking Centre in Mental Health), Madrid, Spain; ^8^Immune System Diseases-Rheumatology, Oncology Service an Internal Medicine, University Hospital Príncipe de Asturias, (CIBEREHD), Alcalá de Henares, Spain

**Keywords:** major depressive disorder, malnutrition, epigenetics, S-adenosylmethionine, micronutrients, omega 3 polyunsaturated fatty acids, pre/probiotics, mineral deficiency

## Abstract

Major depressive disorder (MDD) is a complex, multifactorial disorder of rising prevalence and incidence worldwide. Nearly, 280 million of people suffer from this leading cause of disability in the world. Moreover, patients with this condition are frequently co-affected by essential nutrient deficiency. The typical scene with stress and hustle in developed countries tends to be accompanied by eating disorders implying overnutrition from high-carbohydrates and high-fat diets with low micronutrients intake. In fact, currently, coronavirus disease 2019 (COVID-19) pandemic has drawn more attention to this underdiagnosed condition, besides the importance of the nutritional status in shaping immunomodulation, in which minerals, vitamins, or omega 3 polyunsaturated fatty acids (ω-3 PUFA) play an important role. The awareness of nutritional assessment is greater and greater in the patients with depression since antidepressant treatments have such a significant probability of failing. As diet is considered a crucial environmental factor, underlying epigenetic mechanisms that experience an adaptation or consequence on their signaling and expression mechanisms are reviewed. In this study, we included metabolic changes derived from an impairment in cellular processes due to lacking some essential nutrients in diet and therefore in the organism. Finally, aspects related to nutritional interventions and recommendations are also addressed.

## Introduction

Major depressive disorder (MDD) is a complex and multifactorial neuropsychiatric disease occurring as a result of multiple changes in the brain and the entire organism (1). The World Health Organization (WHO) ranked MDD as the third cause of the burden of diseases globally in 2008, projecting that by 2030, it will become the leading one (2). The estimated global prevalence of MDD was about 4.7% with an annual incidence of a 3% (3). However, the global burden of MDD has been increasing during the last years, specially due to the coronavirus disease 2019 (COVID-19) pandemic (4). Likewise, the risk for suffering from MDD is 2-fold higher in woman than in men, showing some particularities in the underpinning biological mechanisms (5), and although the prevalence may vary across ages, this condition may appear virtually at any stage of life (6). Furthermore, MDD entails devastating individual and socioeconomic consequences. For instance, the risk of suicide is notably higher in subjects with depression, especially for young men (7). In the same manner, being diagnosed with MDD is also related to an increased risk of suffering from cardiovascular death, functional impairment, disability, and decreased workplace productivity and absenteeism (8). Collectively, these events lead to huge economic losses, which may also be attributed to the cost of their derived medical treatments, that frequently are not enough to aid neither in the clinical management of depression nor in their complications (9, 10).

In general terms, psychiatric disorders are considered multifactorial conditions resulting from an interplay of genetic and environmental factors that drive to a set of molecular, cellular, circuitry, structural, and functional changes in the brain (11). In this sense, there are no single gene identified as a causative agent of any psychiatric disorder, including MDD, and there has been a recognition in the need of different environmental factors to explain the onset and progression of these conditions (12, 13). Epigenetics are the central link between genetics and environmental agents, as it modulates the expression of critical genes products under certain environmental conditions (14). Hence, growing efforts are being placed to influence in the epigenetic mechanisms involved in the development of different psychiatric disorders, including MDD (15, 16). Diet is a promising modulator of several epigenetic mechanisms in the entire organism, also in the brain, where it modulates the expression of several genes involved in the function of this organ (17–19). The patient with depression is frequently co-affected by malnutrition. It is not easy to assure if depressive status leads to bad dietary habits and hence micronutrient deficiencies or if those deficiencies are part of the onset of MDD. What it has been observed is that these patients are likely to lose weight involuntarily or suffer deficiencies of essential nutrients (20). Thus, the aim of this review is to collect the available evidence of the epigenetic origins of MDD, concretely evaluating the actions of diet in the onset and development of MDD. Furthermore, we will focus on the translational opportunities derived from this knowledge, and future directions to follow to unravel these complex interactions.

## Epigenetic Basis Of MDD

### Is MDD an Epigenetic Malady?

To answer this question, starting with the definition of epigenetics is a need. This term refers to “the changes in gene function that cause their activation or deactivation without any alteration in the DNA sequence” [National Human Genome Research Institute (21)]. In this context, multifactorial diseases such as psychiatric disorders emphasize the importance of stress-related and environmental factors, which have pointed more prominence in the etiology than genetic factors. Discordances among identical twins studies have justified that frequent exposure to environmental stressors prompts stable changes (i.e., epigenetic marks) in the gene expression with a consequent impact on neuronal functions and, therefore, behavior (22).

The modifications that cause those events can be mediated by DNA methylation, histone modification, and also the expression of signaling non-coding RNAs, mainly represented by long-noncoding RNAs (lncRNAs) and microRNAs (miRNAs) (23–25). That epigenetic regulation can occur not only from nervous system development but also in the mature brain with long-lasting effects and the possibility to be heritable for multiple generations (26). These changes can lead to maladaptive neuronal plasticity, poorer resilience to stress, depressive mood, and different response to antidepressants (27). Recent work reviews have denoted the lack of information regarding the validation of depression-associated epigenetic modifications due to the short age of this field of study, the small sample size of patients, and the difficulties to study functioning changes in alive brains instead of postmortem (23), although there are some epigenetic markers that could be studied in serum and body fluids such as miRNAs (25). Besides, sometimes it is not possible to establish a clear causality of the epigenetic findings because of the difficulty of replicate the experimental results from animals to humans (28).

Systematic reviews have identified so far several alterations in the expression pathways of genes such as brain-derived neurotrophic factor (BDNF), oxytocin receptor (OXTR), nuclear receptor subfamily 3 group C member 1 (NRC31), sodium-dependent serotonin transporter (SLC6A4), FK506 binding protein 5 gene (FKBP5), spindle and kinetochore-associated complex subunit 2 (SKA2), leucine-rich repeat and Ig domain containing 3 (LINGO3), POU class 3 homeobox 1 (POU3F1), a transcriptional repressor of myelin-specific genes, and integrin beta-1 (ITGB1), effect on cell adhesion and several viruses receptor; in the signaling of glucocorticoids, serotonin, and neurotrophins (mainly, BDNF pathways) among others, all these are associated to traumatic events such as childhood maltreatment (29–31). This knowledge has provided a field to understand the long-term effects of adverse life events and aberrant gene expression related to MDD pathogenesis and psychiatric disorders in adulthood in general (32, 33).

Chronic stress has been reported to have pleiotropic effects altering selectively DNA methylome and chromatin compaction, involving mood and even pain perception (34). For these reasons, many authors have argued about an epigenetic basis for the onset of psychiatric disorders, so, it can be affirmed that depression is an epigenetic malady as queried.

### Epigenetic Marks Described in Patients With MDD

#### Histone Modifications

Histones are pivotal structural elements of the chromatin in eukaryotic cells together with DNA and non-histone proteins. There are five major groups of histones, namely, H2A, H2B, H3, H4 (considered core histones and implicated in the formation of nucleosomes with DNA), and H1/H5, involved in the link of multiple nucleosomes and further DNA packaging (35). Far beyond its structural relevance, histones closely impact chromatin function and dynamics, affecting the chromatin expression due to the presence of specific histone variants (i.e., H2A.X, H2A.Z, macroH2A, H3.3, and CENP-A) or through posttranslational modifications (36). In this context, cumulative evidence is supporting the role of the histone variant H3.3 in the pathogenesis of MDD. Specifically, H3.3 dynamics is activated in the depressed human nucleus accumbens (NA) and in response to chronic social defeat stress in mice, whereas the use of antidepressants prevents H3.3 dynamics, limiting its negative effects (37). More data are available regarding the role of posttranslational modifications of histones. Histones are basic proteins particularly rich in lysine and arginine, also presenting other critical amino acids such as serine and threonine, which are prone to suffer from different modifications. These modifications include acetylations/deacetylations (at lysine), methylations/demethylations (at lysine and arginine), phosphorylations/dephosporylations (at serine or threonine), or ubiquitylations/deubiquitylations (36). Of them, histone methylation and acetylation are the most important posttranslational modifications implicated in the pathophysiology of MDD. The upregulation or downregulation of genes depend on the brain region and histone modification involved. Sun et al. (38) summarized many prodepressive epigenetic changes of this type: in the NA, there is an increase of histone deacetylases (HDACs), HDAC2 expression, and decrease of HDAC5 and H3K9me2 (demethylation of lysine 9 of histone 3); in the hippocampus, there is a decrease in H3/H4 acetylation and H3K9me3 and increase in the HDAC activity and HDAC5 expression; and in peripheral blood also aberrant epigenetic marks are found, such as increasing levels of HDAC2, 4, 5, and SIRTs (1,2,6) (sirtuins, a group of enzymes closely related to HDACs) (30). Histone lysine methylation affects neurons of the central nervous system (CNS), being considered a critical regulator of complex processes such as long-term memory formation and behavior (39). HDACs also alter Rac1 transcription (RAS superfamily of small GTP-binding proteins) in NA, leading to an impairment in the synapsis interfering in social defeat stress, social avoidance, and anhedonia (40).

There has been arising promising therapeutic approaches targeting HDACs and other hallmarks in pharmaco-epigenomics of MDD, which may offer broader effectiveness. For instance, HDAC inhibitors can upregulate neurotrophic factors, allowing an enhanced neural plasticity and exerting antidepressant-like behavior (41). In the same manner, HDAC inhibitors can attenuate the neuroinflammation being now considered as an anti-inflammatory treatment (42). Antidepressants have shown to reduce levels of HDAC4 recruitment along with an increased transcriptional activity of glial cell-derived neurotrophic factor (GDNF) in mice (43). Many preclinical models of HDAC inhibitors such as sodium butyrate, alone or in combination with antidepressants, have shown better antidepressant responses (44). These benefits can also be due to the modulation of this drug of DNA methylation, upregulating the enzyme ten-eleven translocation methylcytosine dioxygenase 1 (TET1), resulting in BDNF overexpression in the prefrontal cortex (45).

#### DNA Methylation

Many studies have been focusing on DNA methylation in CNS or peripheral tissue. Despite the small sample sizes and low replicative results, omics data and candidate-gene approaches (many about SLC6A4, BDNF, and NR3C1) are on the way to answer more etiological questions (46). Interestingly, maternal stress during pregnancy is key for the fetal epigenetic programming. Part of the maternal cortisol can pass to fetus and consequently, increase the expression of DNA methyltransferase 3a (DNMT3a) and then increase DNA methylation at the promoter region of converting active-to-inactive cortisol enzyme 11β-hydroxysteroid dehydrogenase type 2 (HSD11B2), leading to a lower expression of this enzyme at the fetal cortex and increasing the susceptibility to stress in later life (47). Not only emotional stress but also other stressors such as nutritional restriction can alter highly GC-rich zones in promoter core and downregulate HSD11B2 expression in placenta (48). All in all, the glucocorticoid exposure in the intrauterine environment is key for the DNA methylation of stress response genes, including HSD11B2 and also NR3C1. Some researchers have conducted studies to observe the joint contribution of these genes' expression in newborns neurobehavior, describing different phenotypes, including babies with low NR3C1 methylation but high HSD11B2 methylation had lower excitability scores; babies with high NR3C1 methylation but low HSD11B2 methylation had more asymmetrical reflexes; and lastly, those with high DNA methylation in both genes had higher habituation scores (49). These statements are in agreement with what several scientists have hypothesized as “the fetal origin of diseases” from the epigenetic reprogramming, in this case, “the fetal origin of psychopathology” (50). Although there is still little support from observational studies, there is much consideration about fetal origins of mental health in later life. Maternal depression in pregnancy is considered a serious public health concern, being estimated to increase the depression risk to a 4-fold in the offspring (28). Some evidence has also suggested that several infections and their inflammation during pregnancy may cause injuries in neurodevelopment and then increase the risk for autism spectrum disorder and depression (51).

Furthermore, early childhood stressful experiences have been also linked to changes in gene expression of hypothalamic-pituitary-adrenal axis (HPA), glucocorticoid signaling pathway (i.e., NR3C1 and FKBP5), neurotrophic factors (i.e., BDNF), serotonergic neurotransmission (i.e., SLC6A4), estrogen receptors, and arginine vasopressin, among others. In this line, it has been questioned if these early adverse events establish the features of our personality (52). The link between early-life social stress and different methylation patterns has been studied in animal models. High methylation by DNMT3 in CpG islands from promoter regions entails the downregulation of serotonin and its transporter (i.e., SERT) together with the upregulation of monoamine oxidase A (MAO-A) and tryptophan hydroxylase 2 (TPH2). All these genes are part of the process of brain development, stress response, and emotional control (53). Moreover, maltreated children have shown hypermethylation in the promoter region of GR gene NR3C1 compared with non-maltreated children, entailing transcriptional silencing. These results corresponded with emotional negativity, ego under control, and more externalizing behavior with depressive symptoms (54, 55).

Long-lasting affection of the HPA axis function has implications for health and well-being in later life. Those environment challenges make changes in brain plasticity, neuronal function, and behavioral adaptation to neuropsychological stress in MDD (56). The dysregulation at this axis is generally accepted to be a consequence from the chronic and exacerbated exposure to glucocorticoids, disturbing already mentioned signaling/levels (57). HPA-dysregulated functioning also entails NRC1 and SLC6A4 hypermethylation, explaining the worse reactivity to stress and disrupted serotonin transport in MDD (58). Either early or later, events that happen throughout our lives may have a long-lasting impact on behavior, bringing a maladaptation that results in changes at limbic regions such as the hippocampus and amygdala. *Reul*'s research group studied these processes and found a link in ERK-MAPK signaling pathway with c-Fos induction, histone H3 acetylation, and DNA methylation at promoter locations. In addition, they concluded that gamma-aminobutyric acid (GABA) could control the different response to such psychological stress and shape those epigenetic changes *via* “local GABAergic interneurons and limbic afferent inputs” (59). The synaptic activity *via* neurotransmitter receptors regulate these epigenetic markers that underlie learning and memory (60), whose impairments are serious incapacitating symptoms in MDD (61).

Whether inherited or acquired, discoveries in non-Mendelian biology demonstrate that epigenetic markers offer new insights in the deeper understanding of complex multifactorial psychiatric disorder of MDD. It is one more variable for MDD multiparametric equation, without forgetting other cumulative effects such as accompanying DNA sequence polymorphisms (62).

#### Noncoding RNAs

**Figure 1:**
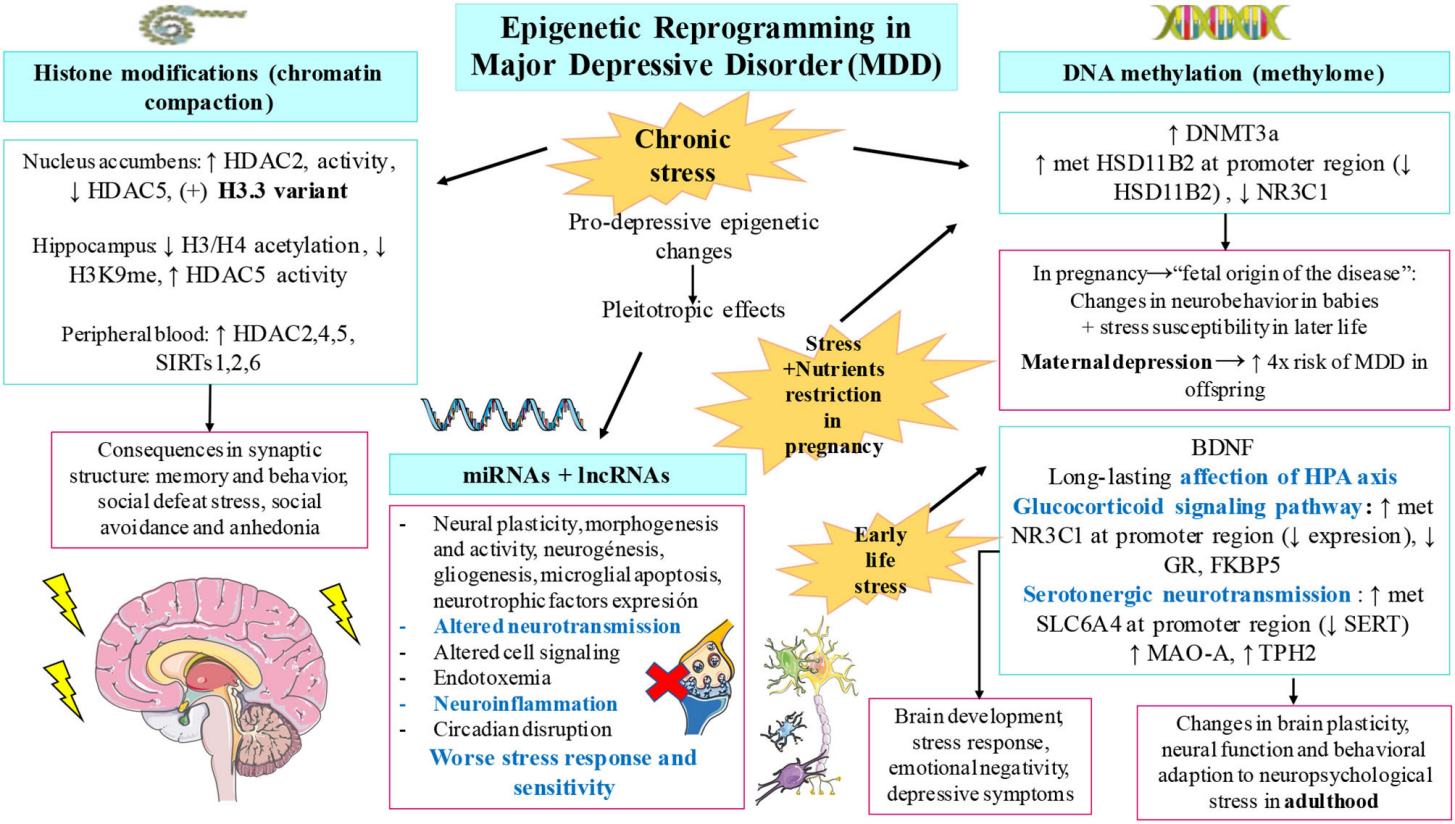
Compiled evidence related to epigenetic changes involved in major depressive disorder (MDD). As shown, chronic stress, early-life stress, and prenatal stress accompanied with nutrient restriction are pivotal drivers of the epigenetic modifications observed in patients with MDD. These changes include histone modifications, altered DNA methylation patterns, and the dysregulation of non-coding RNAs including microRNAs (miRNAs) and long non-coding RNA (lnRNA). Some of the most relevant findings collected in this manuscript for each modification are summarized. These variations lead to important alterations in the brain synapsis, anhedonia, social defeat stress, neurodevelopment, brain plasticity and function, and many other depressive symptoms.

Other novelty epigenetic malleable regulators include non-coding RNAs. First, miRNAs are small molecules carried in vesicles, which are implicated in cell-cell communication, being crucial for neuronal morphogenesis, activity, and plasticity, besides having prominent systemic effects. During the last decade, numerous miRNAs with pleiotropic effects have been identified to be involved in several processes that concern MDD pathogenesis, including neuroinflammation, endotoxemia, microglial apoptosis, altered neurotransmission, worse stress response and sensitivity, altered cell signaling, and circadian disruption. Most of these effects are reviewed and summarized by Ortega et al. (25). In light of the evidence, it is undeniable that miRNAs are key elements implicated in MDD pathogenesis, representing promising therapeutical targets (63). In the similar manner, *lncRNAs* are similar to non-coding RNAs with important signaling and epigenetic actions. In fact, it is now known that they play a synergistic effect with miRNAs. Apparently, these lncRNAs are highly expressed in the brain, and their dysregulation shapes negatively neural stem cell maintenance, neurogenesis and gliogenesis, HPA axis, neurotransmission, neuroinflammation, neurotrophic factors expression, stress responses, and neural plasticity, being currently considered new biomarker candidates of MDD (24, 64, 65). Compiling written evidence about miRNAs and other epigenetic mechanisms in MDD is summarized in Figure 1.

## Nutrition, Epigenetics, And Mdd: Is There A Link?

The question that comes next is if those epigenetic marks lead to a worse nutritional status, if bad dietary habits lead to acquire different gene expressions, or both may be actually occurring. First, it would be helpful to describe a general picture from the nutritional status in a patient with depression and, afterward, focus on the effect of specific deficits and metabolic impairments in the context of malnutrition-related MDD.

### Malnutrition in the Patient With Depression

Currently, it is broadly accepted that there is a link between MDD and malnutrition. Actually, the allowed evidence is not based on standardized methods of the nutritional assessment in the patient with depression, what complicates the drawing of conclusions beyond known micronutrient deficiencies. Although clinical practice nowadays includes the recommendation of supplementation intake to supply certain common deficiencies, mainly vitamin D and omega 3 polyunsaturated fatty acid (ω-3 PUFA), it is also unusual to find observational studies of patients with MDD who have undergone a nutritional assessment. Considering the nutritional status in the management of a patient with depression is relatively new, and, over the last 10 years, surveys, anthropometric, and biochemical measures have started to be used for studying depression in the elderly population both at developed and in developing countries.

#### Nutritional Assessment in MDD

First, *Mini Nutritional Assessment* (MNA) questionnaire and the *Geriatric Depression Scale* (GDS) have been strongly associated. Malnourished geriatric patients or patients at risk of malnutrition have higher risk of suffering from MDD. These studies have been useful for determining the prevalence and severity of MDD and its relationship with malnutrition (66); moreover, a worsening of the nutritional status is also observed in old subjects with depression (67). An evaluation of nutritional status and GDS in community-dwelling elderly people have also been valuable for an early identification of non-diagnosed depression in individuals with nutritional disorders (68). MNA is considered a useful tool for monitoring patients of any age, at risk of undernutrition, which is more common in MDD than overnutrition (69). Thus, there has been a growing awareness of the importance of this fact, and every time, more hospitals are contemplating the role of nutritionists to reduce health costs. In poor infrastructure areas, it has been a cost-effective measure to warn about the struggles in the quality of life of their population, always finding an association between MDD and malnourishment (70–72).

Furthermore, some studies have introduced anthropometric parameters where the tendency showed abdominal obesity or higher amount of abdominal fat in patients with depression (73). Systematic reviews have found solid data in the association between depression, anthropometric parameters, and body image in all included studies, notwithstanding the different statistical methods employed. It is frequent to find among individuals with depressive symptoms: women perceiving their body bigger than reality and men perceiving themselves as underweight idealizing larger bodies (74). Recent studies have identified anthropometric parameters as risk markers (e.g., waist-to-hip ratio) for suicide ideation and severity of illness in women with postnatal depression (75).

Furthermore, to assess concretely nutritional deficiencies, questionnaires about food intake and biochemical markers measurements are the norm. Metabolic parameters include low hematocrit, low high-density lipoprotein cholesterol (HDL-C), and high triglyceride levels in patients with depression (76). More precisely and above mentioned, concrete groups of essential micronutrients are often much lower in these patients. Patients with MDD lack vitamin B consumption, especially cobalamin (B12) and folate (B9) (73, 77), as well as pyridoxin (B6) (78). Low vitamin D serum levels are positively associated with depression (79), although insufficient dietary intake is not the only cause, being little outdoor exposure to sunshine is more relevant (80). Moreover, low circulating ω-3 PUFA has been linked not only to MDD but also to preterm birth and prenatal depression associates with preterm birth (81). Low intake of marine ω-3 PUFA, especially docosahexaenoic acid (DHA), increases the risk of many mental issues, besides MDD, suicidal ideation, bipolar disorder, autism, and attention deficit hyperactivity disorder (82). Eventually, there is an insufficient intake in minerals, commonly calcium, iron, magnesium, and zinc (83). The list is even longer in the case of women, but not men, with depressive symptoms according to recent studies, including also potassium, phosphorus, and copper (84). All these nutrients are vital for monoamines synthesis, neuroinflammation control, neuroprotection, and the synthesis of growth factors (85).

To address these deficiencies, it is of note to be aware about changing patients' nutritional behavior and the diet composition prior to the onset of MDD and during the course. Food patterns heading depression are kept in the course of the disorder: poor appetite, skipping meals, and sometimes, a dominant preference for high-sugar foods (emotional eating) (86). There is recent research establishing relationship between macronutrients and depression through surveys in big samples of patients. The results showed a significant low proportion of protein intake associated with the prevalence of MDD (87). Food frequency questionnaires have shown the important issue of quality and quantity of protein intake, being low consumption of protein-rich foods such as milk, and legumes significantly associated with higher mean scores of depression and anxiety symptoms (88, 89). Diet is also known to be the greatest shaper of gut microbiota, and this complex “organ” is even involved in the synthesis of vitamin B and may affect host vitamin B usage (90, 91). In fact, B12 intake or status is associated with microbial diversity, relative abundance of bacteria, and short chain fatty acids (SCFAs) production (92). Then, the underlying characteristic gut dysbiosis of MDD might not supply those vitamins apart from dietary sources.

#### Comorbidities and Eating Disorders in MDD

Furthermore, several comorbidities associated to dietary habits and intestinal problems frequently co-occur with high prevalence in patients diagnosed with MDD and vice versa. In this context, there is a high co-occurrence of inflammatory bowel disease (IBD) with MDD and/or anxiety, and observational prospective studies denote a high incidence of MDD in patients with diagnosed IBD (93). There is bidirectionality: on one hand, this is observed to be due to poor self-management, which leads to disease chronicity (94), but in contrast, there is evidence that the course of IBD is worse in patients with depression, being the corticosteroid treatment able to induce the psychiatric symptom onset (95). Systematic reviews explain that patients with IBD had 20% prevalence rate of anxiety and 15% prevalence rate of depression until 2016 (96), but the rising prevalence of both kind of maladies has changed numbers until 2021, being 33 and 25% currently, respectively (97). Some empirical studies demonstrated that the symptoms of anxiety/depression are related to more aggressive forms of IBD, emphasizing that psychiatric treatment is also vitally important to ameliorate the prognosis of IBD (98, 99). Fortunately, some statistical data have been reunited identifying the selectively protective role of certain antidepressants for Crohn's disease and ulcerative colitis, including monoamine oxidase inhibitors, serotonin norepinephrine reuptake inhibitors, selective serotonin reuptake inhibitors, serotonin modulators; and tricyclic antidepressants (100).

Metabolic disturbances can also occur after the onset of MDD or before, including obesity, type 2 diabetes mellitus, or metabolic syndrome. Some evidence alleges that metabolic signaling of leptin and ghrelin might play a great part in the dysregulation of mood (101). In this line, metabolic dysregulation seems to go hand in hand with chronic stress and mental disorders. High leptin levels and binge eating and emotional eating are positively associated. This hormone is involved in reward circuits whose maladjustment leads to pathological eating behaviors (102). High daily cortisol is sometimes related to hyperleptinemia, making individuals more vulnerable to stress-induced eating (103). Moreover, emotional eating is not the norm; especially in late-life depression, there is a high tendency to appetite loss and involuntary weight loss (104). In scientific literature, we may find two subgroups of MDD according to appetite changes; these are the terms, “depression-related increases in appetite” and “depression-related appetite loss.” The first one is associated with a hyperactivation of mesocorticolimbic dopamine reward circuits, whereas the latter is associated with a hypoactivation of mid-insular cortex implicated in interoceptive and homeostatic signaling (105).

All in all, overconsumption does not guarantee vitamins, minerals, and other essential nutrients herein discussed and definitely neither does undernutrition. A consequent maladaptation from inadequate dietary habits entails metabolic changes, which are associated to severity of symptoms and other comorbidities. The turning point that comes next is to find the link between those deficiencies and subjacent epigenetic molecular mechanisms, which take part in the basis of MDD pathophysiology.

### Epigenetic Roles of Diet and Nutritional Status in MDD

Diet is being considered an environmental epigenetic factor, with nutritional epigenetics being the science that intends to explain the effects of nutrients on gene expression and metabolism (106). This field aims to explain the association of suboptimal nutritional environment as a driver of potential adult-onset chronic illnesses due to shifts in genome functions (107). *Landecker* reviewed and argued that some genomes immersed in food molecules might be more susceptible to epigenetic lability than others predisposing them to a determined susceptibility to disease (108). On the one hand, some bioactive food compounds are able to exert protective properties, and in contrast, recently, it has been studied that some components from western-type diets, ultraprocessed food, and their lack of essential nutrients also modulates negatively epigenetics machinery (109, 110).

#### Diet as Lifestyle Habit

An adequate nutrition is essential during development, in prenatal and postnatal periods of life, what in fact, it is called “window of opportunity,” the first 1,000 days from pregnancy to 2nd birthday (111, 112). Epidemiological studies assure that maternal nutrition in development provides a wide variety of epigenetic changes being key for susceptibility to disease phenotypes in later life (113). A great part of modifications occurs during early embryonic and primordial cell development, although what we have not completely understood is their potential echo in this “later life” (114).

The underlying biological mechanisms have been deeply watched in animal models. What evidence says is that inadequate maternal nutrition patterns, either undernutrition or overnutrition, exert alterations in DNA methylation mechanisms in the hypothalamus, concretely in pathways involved in energy homeostasis, with an echo in adulthood. A maintained protein restriction in postnatal development was related to an immature hypothalamus as well (115–117). Other findings related to high-fat diet consumption during pregnancy were the upregulation of dopamine reuptake transporter (DAT) in the ventral tegmental area, NA, and prefrontal cortex and a downregulation of DAT in the hypothalamus. These data result from changes in DNA hypomethylation at promoter regions of DAT, and the association observed was long-term alterations in the expression of dopamine and opioid-related genes, as well as changes in food behavior (preference for more palatability) (118). Conversely, undernutrition is associated with hypomethylation of hypothalamic GR without changes in the hippocampus, contributing to altered energy balance regulation in the offspring (119). Nevertheless, even in adult life, maladaptive embryonic and perinatal epigenetic changes can potentially be reversed or attenuated; it is known that epigenetic marks are really plastic (120). Although the epigenome is shaped by nutritional states, even those more stable are malleable by implementing a different diet (121). This can be possible with nutrient-rich bioactive foods or with food-based bioactive components (e.g., polyphenols, ω-3 PUFA, resveratrol, curcumin, and green-tea compounds, among many others), opening the gate to prevention and treatment of multifactorial non-communicable diseases and mental disorders, including MDD (122) as it will be addressed later. Many dietary components have the power to influence pathways that change DNA methylation patterns, and the evidence has demonstrated the biochemical routes between the diet quality and mental health (123). Maintaining an environmental stressor such as western-type diets, full of ultraprocessed foods, denotes imbalance of macronutrients and deficiencies in micronutrient levels, as above reported in malnutrition associated with the patients with depression. These have been correlated with alterations in behavior, but the consequent maladaptation of the epigenome has not been elucidated yet in the context of MDD pathophysiology.

Nutrients are needed to accomplish biological functions. One of the epigenome-diet hallmarks involves methionine and folate from the diet, whose metabolism gives rise to S-adenosylmethionine (SAMe), considered as the universal methyl donor for DNA and histone methylation reactions. Nutrient availability will provide SAMe, and this will heavily control gene expression (124). Affecting SAMe metabolism and deficiencies in B6, B9, B12, and zinc (which act many times as cofactors for enzymes and methyl donors) are correlated to high homocysteine levels, a risk factor traditionally associated with multifactorial inflammatory diseases and now also with MDD, psychosis, suicide ideation, or alexithymia (125–127).

Psychiatric involvement of B12 deficiencies with high homocysteine and methylmalonic acid denotes memory impairment, depression, and other manifestations (i.e., mania, psychotic symptoms, and obsessive compulsive disorder) (128–131). For these reasons, B12 levels were proposed to be assessed in neuropsychiatric disorders and neurodegenerative diseases and advised to be evaluated with treatment resistant disorders and certain risk factors that link malnutrition with MDD, including alcoholism, advancing age with neurological symptoms, anemia, intestinal problems, and malabsorption or strict vegetarian diets (132, 133). These causes would also explain the associated dysbiosis that, due to cobalamin deficiency, destabilizes microbial communities, who would also not be able to produce microbial B12 (134, 135).

Low levels of B6, B9, and B12 are shown to affect methylation levels of redox-related genes. This has been observed in NUDT15 (Nudix hydrolase 15, a hydrolase of nucleoside diphosphates) and TXNRD1 (thioredoxin reductase 1) hypermethylation (31, 136). Thus, the role of oxidative stress of these vitamins is crucial for brain protection (137, 138). In addition, lacking these essential vitamins for neuronal function affects monoamine oxidase production and the repair of phospholipids (139). This could be extrapolated to the reported neurotransmission impairments first due to imbalanced neurotransmission synthesis and second due to damage at axonal and soma membranes (128, 140). These symptoms are in concordance with co-deficiencies of ω-3 PUFA, especially DHA, which is important for neuronal membrane fluidity and neurotransmitter release (82).

Moreover, low protein intake entails scarcity of essential amino acids such as valine, leucine, isoleucine, lysine, phenylalanine, tyrosine, arginine, histidine, and tryptophan, which are necessary precursors for neurotransmitter and neuromodulator synthesis (141–143). For instance, phenylalanine and tyrosine are two essential precursors for the biosynthesis of dopamine, norepinephrine, and epinephrine (144). For its part, vitamin D deficiency is one of the most repeated manifestations in MDD (145), and the reasons are not only subjacent an insufficient dietary intake but also an insufficient outdoor exposure to sunshine (80). The clinical relevance of these observations is known, thanks to preclinical models that have identified their immunomodulator and neuromodulator roles, with protective effects for oxidative stress as well (146). It is known that vitamin D is key for the proper development of dopaminergic neurons and the expression of GDNF (147), and now many vitamin D receptors (VDRs) are found in the substantia nigra, where the enzyme 1α-hydroxylase (CYP27B1) converts it to its active form (148, 149). VDR and CYP27B1 genes can become hypermethylated at promoter regions becoming silenced, and also VDR protein when meeting its ligands can establish contact with histone demethylases, reconfiguring chromatin modeling (150). Vitamin D also has the ability to exert potent antioxidant effects that ease DNA repair, defense against infections, and protection from oxidative stress-related protein oxidation (151).

Notably, many nutrient-related links that may alter MDD pathophysiology have overlapping etiology aspects with neurodegenerative diseases such as Alzheimer's and Parkinson's diseases (152). A deep understanding of these diet-related epigenetic shifts becomes necessary, highlighting complementary branches such as nutritional neuroscience and nutritional psychology for the integrative study of MDD aiming to improve prognosis or prevent the onset of MDD and neurological impairments.

#### Diet in Microbiota Neuromodulation

Regarding food consumption, much research has focused on the effects of diet and lifestyle on epigenetic reprogramming. Although some dietary components may exert some direct epigenetic effects, prior studies have noticed a critical interplay between diet and gut microbiota in the epigenetic profile of the host (153). As we know, the microbiota-gut-brain axis is a bidirectional system, and considering diet as the greatest shaper of gut microbiome, microbial metabolite production is undeniably diet-dependent. For example, it is known that tryptophan levels allow microbial serotonin synthesis (154), or dietary fiber allows GABA, norepinephrine, tryptamine, and dopamine microbial synthesis (155). Thus, we emphasized that diet potentially modulates microbial contributions to the neurotransmission system in the human gut.

Moreover, mainly, dietary fiber drives to the production of SCFAs using the gut microbiota, acting as HDAC inhibitors, and regulating DNA methylation, histone modifications, and chromatin restructuring to alter gene expression (156). One proven effect of the epigenetic and antidepressant action of SCFAs was described in a mice model. In this study, the inhibition of HDAC by SCFAs led to the hyperacetylation of histones H3/H4 resulting in an increased BDNF expression (157). SCFAs production is even lessened due to the co-deficiencies of certain vitamins, which exert important roles in intestinal homeostasis too. *Pham et al*. reviewed and summarized the positive effects of adequate levels of vitamins on gut microbiota health: vitamins A, B2, D, E, and beta-carotene increase relative abundance of commensals; vitamins A, B2, B3, C, and K increase diversity; vitamin D boosts diversity; and vitamin C, B2, and E enhance SCFAs production (158).

#### Diet and miRNAs

In this study, it faced an emerging and challenging research area. Some disease-specific miRNAs profiles have been associated to MDD, and the expression of these molecules can be affected by dietary factors (25). Posttranscriptional regulation through miRNAs depends on sensory functions from carbohydrates, proteins, fat, vitamins, minerals, and fiber (159). Deficiency or excess of certain nutrients at any age, from embryonic development to senescence, has been correlated to disease onset. The mechanisms exerted from nutrient absorption are the expression of different profiles of miRNAs, which will target other components from epigenetic machinery, affecting DNA methylation and histone modification and then the gene expression at different levels: immunophenotypes and inflammation/immunoregulation balance, cardiovascular health, insulin sensitivity/resistance, and muscle health (160). For instance, the research says that over intake of fat combined with low vitamin D intake leads to dyslipidemia by impairments in miRNAs expression, which is also related to macrophages polarization in associated digestive comorbidities mentioned such as IBD (160, 161).

More recently, in the past decade, it was discovered that some food-derived miRNAs (xenomiRs) from plant and animal sources affect individual's gene expression, suggesting a cross-kingdom communication (162, 163). However, several studies have found difficulties to distinguish most dietary from endogenous miRNAs and disparity of results in this new field of food science. In this sense, there are already hypothesis to prove, for instance, for checking how miRNAs-deficient diet may influence health and disease (164). Nonetheless, milk exosomes and their miRNA cargos have been found in different mammalian organs (e.g., liver, spleen, brain, and intestinal mucosa) (165), and exogenous plant miRNAs have been found in mammalian tissues targeting low-density lipoprotein receptor adapter protein 1 (LDLRAP1), decreasing low-density lipoproteins (LDLs) in plasma (166). Some authors have also suggested that gut microbiota status may ease or not xenomiRs bioavailability through exosome like nanoparticles at the same time that xenomiRs may modulate microbiome functions (163). There are interesting studies about it, for example, ginger exosomes are mainly absorbed by *Lactobacillus rhamnosus* and promote IL-22 production improving intestinal barrier (167).

A proposal of further research would be interesting for the link between miRNAs profiles that have been already identified in MDD and if certain dietary behaviors contribute to their different expression having an impact on the pathophysiology.

## Translational Approaches: Targeting The Epigenome Through Diet For The Patient With MDD

In the last section, the nutritional status of subjects with depression and the epigenetic consequences were reviewed. In this study, we will discuss the most relevant studies regarding the benefits from receiving nutritional support and how this may modulate the epigenetics of brain and the body of individuals with MDD.

First, it should be mentioned here the main strategies currently used in the clinical management for MDD. As mentioned above, MDD is presented by the diagnosis of at least one of the two main criteria, namely, loss of interest (i.e., anhedonia) or depressed mood and ≥4 somatic and non-somatic items (such as loss of appetite, insomnia, and low energy), minimum presented in a period of 2 weeks (168). These makes MDD a very heterogeneous disorder, with many therapeutic difficulties. For instance, notwithstanding the use of antidepressants is widely accepted for the therapy of MDD, cumulative evidence supports that the use of antidepressants and current clinical guidelines may not be sufficient for an important part of subjects with depression, especially for those with severe symptoms, that exert worse clinical outcomes despite receiving greater intensity of treatment (169). Besides, ~30% of people with MDD are resistant to conventional treatment (170), and there is still a big debate about if the main benefits of antidepressants are due to their action or if conversely, it may be attributed to the placebo effect (171, 172). This could be due to the fact that many antidepressants target serotoninergic and monoamine neurotransmission, which traditionally has been claimed as the major pathophysiological mechanism of MDD (173, 174). However, as previously described, currently, it is widely accepted that MDD is associated with a plethora of additional pathophysiological mechanisms. Because of that, it is necessary to accept the huge difficulties in the clinical management of patients with MDD; there is an urgent need for improving the clinical guidelines and for reviewing multidisciplinary approaches that may bring the maximum benefits to these patients.

In this great context, nutritional interventions can be excellent supportive strategies in MDD. A meta-analysis conducted by Firth et al. (175) including 45,826 participants show that dietary interventions may be of great aid for the prevention and amelioration of depressive symptoms. However, most subjects were not diagnosed with clinical depression, so their conclusions might not be extrapolated to MDD. Recently, an umbrella meta-analysis conducted by Xu et al. (176) has obtained some important results establishing an inverse relationship between different nutritional approaches, group of foods, and nutrients in the prevention and treatment of depression. However, the methodological quality of most of the selected meta-analysis was low or very low, which may also bring some caution with their results. In fact, some studies have argued that most narrative reviews come to strong benefits from dietary interventions in patients with MDD, despite the level of evidence is still inconsistent (177). Then, they conclude that more systematic reviews and objective data are needed before stablishing some conclusions, and we encourage for further research and studies in this field.

Previously, we defined the epigenetic consequences of malnutrition in patients with MDD. As detailed, subjects with depression often exhibited different concerns in their intake of macro- and micronutrients. Then, by modulating the levels of these nutrients, it is possible to address the multiple issues related to their nutritional deficits and overconsumption. Thus, patients with MDD will benefit from two different ways of the nutritional intervention, namely, (1) By limiting their consumption of unhealthy products and nutrients and (2) by addressing the nutritional deficiencies. In this sense, diet has the potential to aid in the clinical management of MDD. It is worthy to mention that there are neither a single nor best option for the general population with depression; conversely, the most important part of a healthy diet is to provide an adequate intake of macronutrients, micronutrients, and hydration to meet the physiological needs of the body (178). Thus, a healthy diet contains a wide variety of foods of nutritional interest, which are crucial for health preservation. Besides, to those foods or part of them with promising actions either in the prevention or as a therapeutic adjuvant of different NCDs are defined as “Nutraceuticals” (179). What evidence seems to support is that there are specific group of foods and nutrients with promising antidepressant effects, most of them included in a healthy dietary pattern such as Mediterranean diet (180–183).

Overall, because of the growing awareness of the critical role of diet in the management and prevention of MDD, we encourage for the development of further studies in this area regarding different dietary approaches and group of foods and nutrients/bioactive compounds with promising benefits for the treatment of MDD, focusing on their epigenetic role as a promising point to consider explaining their positive effects.

## Conclusion

**Figure 2:**
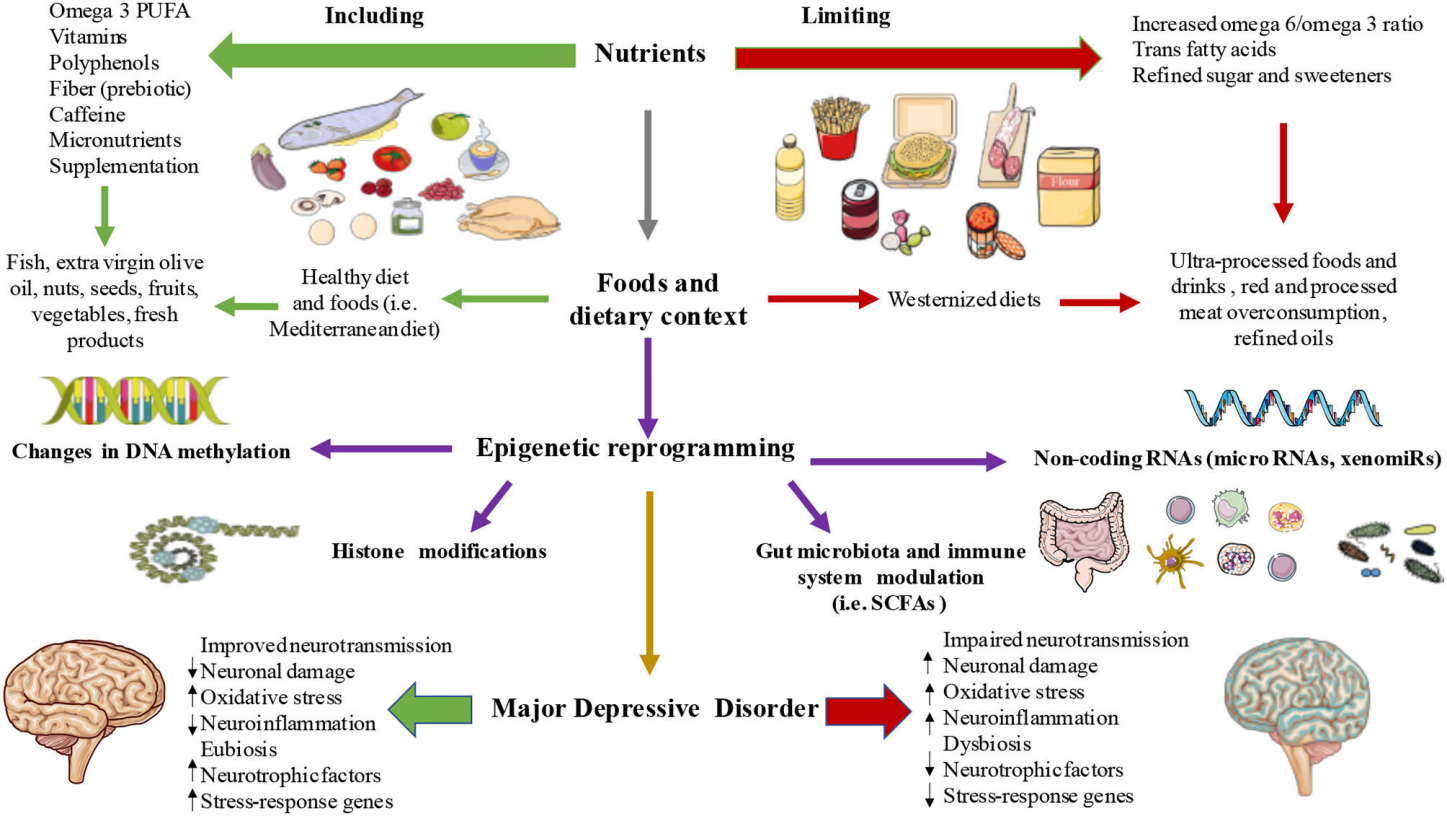
The link between diet, epigenetics, and MDDs. As summarized, a set of nutrients, foods, and dietary strategies are able to influence different epigenetic mechanisms like DNA methylation, histone modifications, non-coding RNAs, and gut microbiota-immune system composition and their products thereby influencing in the pathophysiology of MDD.

The link between nutritional epigenetics and MDD composes a new field of research. A deep understanding of these diet-related epigenetic shifts becomes necessary highlighting complementary branches such as nutritional neuroscience and nutritional psychology for the integrative study of MDD. This review intended to unify different areas of research to serve as a link between malnutrition-related epigenetic changes that seem to be involved in MDD pathophysiology as summarized in Figure 2. Perhaps, not many studies have demonstrated the clear association to determine causality from observational studies, and it is undeniable that more empirical data are needed. However, as we herein followed, the epigenetic role of diet demonstrates that it can alter several neuronal pathways (e.g., DAT, GR, HPA axis, neuronal membrane fluidity, neurotransmission, microbial neurotransmitters synthesis, neuronal damage, oxidative stress, and a long etcetera), that have been studied in the context of MDD pathophysiology, and new advances in clinical trials are demonstrating promising results in the reversion or attenuation of those epigenetic marks. There are misunderstandings yet in the pathophysiology of MDD in general and in the still developing field of associated epigenetic drivers, even more, especially in the knowledge of the relationship between malnutrition-consequent epigenetic markers involved in MDD pathophysiology. Fortunately, we are on the era in which precision medicine, integrative therapies, and the premise “we are what we eat” are gaining stronger echo.

## Author Contributions

All authors listed have made a substantial, direct, and intellectual contribution to the work and approved it for publication.

## Funding

This study was partially supported by grants from the Fondo de Investigación de la Seguridad Social, Instituto de Salud Carlos III (PI18/01726 and PI19/00766), Spain, Programa de Actividades de I+D de la Comunidad de Madrid en Biomedicina (B2017/BMD3804 and B2020/MITICAD-CM), and HALEKULANI S.L.

## Conflict of Interest

The authors declare that the research was conducted in the absence of any commercial or financial relationships that could be construed as a potential conflict of interest.

## Publisher's Note

All claims expressed in this article are solely those of the authors and do not necessarily represent those of their affiliated organizations, or those of the publisher, the editors and the reviewers. Any product that may be evaluated in this article, or claim that may be made by its manufacturer, is not guaranteed or endorsed by the publisher.
